# Cellular and Molecular Pathways in Diabetes-Associated Heart Failure: Emerging Mechanistic Insights and Therapeutic Opportunities

**DOI:** 10.3390/cimb47110886

**Published:** 2025-10-26

**Authors:** Nikolaos Ktenopoulos, Lilian Anagnostopoulou, Anastasios Apostolos, Panagiotis Iliakis, Paschalis Karakasis, Nikias Milaras, Panagiotis Theofilis, Christos Fragoulis, Maria Drakopoulou, Andreas Synetos, George Latsios, Konstantinos Tsioufis, Konstantinos Toutouzas

**Affiliations:** 1 First Department of Cardiology, National and Kapodistrian University of Athens, Hippokration General Hospital of Athens, 11527 Athens, Greece; anastasisapostolos@gmail.com (A.A.); panayiotisiliakis@gmail.com (P.I.); panos.theofilis@hotmail.com (P.T.); christosfragoulis@yahoo.com (C.F.); mdrakopoulou@hotmail.com (M.D.); synetos@yahoo.com (A.S.); glatsios@gmail.com (G.L.); ktsioufis@gmail.com (K.T.); ktoutouz@gmail.com (K.T.); 2 Unit of Structural Heart Diseases, First Department of Cardiology, Medical School, Hippokration General Hospital of Athens, National and Kapodistrian University of Athens, 11527 Athens, Greece; 3 Internal Medicine Department, KAT General Hospital, 14561 Athens, Greece; lilian.anagnostopoulou@gmail.com; 4 Royal Brompton and Harefield Hospitals, Guy’s and St Thomas’ NHS Foundation Trust, London SW3 6NP, UK; 5 Second Department of Cardiology, Hippokration General Hospital, Aristotle University of Thessaloniki, 54642 Thessaloniki, Greece; pakar15@hotmail.com; 6 Cardiology Department, Hippokration General Hospital of Athens, 11527 Athens, Greece

**Keywords:** diabetes mellitus, heart failure, diabetic cardiomyopathy, molecular mechanisms, therapeutic strategies

## Abstract

Diabetes mellitus (DM) is a global health challenge that contributes to numerous complications. As a chronic metabolic disorder, DM leads to persistent microvascular and macrovascular damage, ultimately impairing the function of multiple organ systems. Cardiovascular diseases (CVD), including heart failure (HF), are among the most serious diabetes-related outcomes, accounting for substantial morbidity and mortality worldwide. Traditionally, diabetic HF has been attributed to coexisting conditions such as hypertensive heart disease or coronary artery disease. However, a high prevalence of HF is observed in individuals with DM even in the absence of these comorbidities. In recent years, the phenomenon of diabetes-induced HF has attracted considerable scientific interest. Gaining insight into the mechanisms by which diabetes elevates HF risk and drives key molecular and cellular alterations is essential for developing effective strategies to prevent or reverse these pathological changes. This review consolidates current evidence and recent advances regarding the cellular and molecular pathways underlying diabetes-related HF.

## 1. Introduction

Diabetes mellitus (DM) continues to pose a growing global health challenge, characterized by chronic hyperglycemia resulting from impaired insulin secretion, insulin resistance, or both [1]. Among the organs most affected, the cardiovascular system stands out as particularly vulnerable to the long-term metabolic disturbances caused by diabetes. These effects manifest through an intricate web of microvascular and macrovascular complications [2]. Of particular concern, heart failure (HF) has emerged as a common and often fatal outcome—occurring at markedly higher rates among individuals with diabetes, even in the absence of traditional risk factors such as coronary artery disease or hypertension [3]. This striking pattern challenges the long-held notion that diabetic HF arises mainly as a consequence of ischemic heart disease, underscoring the need for deeper mechanistic insight into how diabetes itself drives myocardial injury and dysfunction independent of overt coronary events [4].

While classical explanations emphasize metabolic derangements, recent research has unveiled a far more complex picture. Over the past decade, accumulating evidence has revealed a multifaceted network of cellular and molecular disturbances that underlie diabetic cardiomyopathy and heighten susceptibility to ischemic injury [4,5,6]. These include mitochondrial dysfunction, regulated forms of cell death such as necroptosis and pyroptosis, maladaptive endoplasmic reticulum (ER) stress responses, disordered autophagy, and diverse epigenetic alterations, all converging to create a hostile myocardial environment [7,8]. In parallel, diabetes disrupts classical neurohormonal and inflammatory pathways, weakening cardiomyocyte resilience and accelerating fibrotic remodeling [9].

Despite significant advances, many reviews fail to synthesize these emerging findings into an integrated understanding of diabetes-associated cardiac injury. This gap limits our ability to appreciate both the pathophysiological complexity and the translational implications of this condition Chronic metabolic disturbances in DM, including sustained hyperglycemia and insulin resistance, contribute to progressive cardiac dysfunction and the eventual onset of HF. While substantial progress has been made in elucidating the pathophysiology of DM-related HF, the precise cellular and molecular mechanisms remain incompletely understood. A deeper understanding of how diabetes accelerates HF development, and the pathways through which it induces key molecular and cellular changes, will be critical for designing targeted therapeutic interventions. This review synthesizes current evidence and recent advances on the cellular and molecular mechanisms underlying diabetes-associated HF.

**Figure 1:**
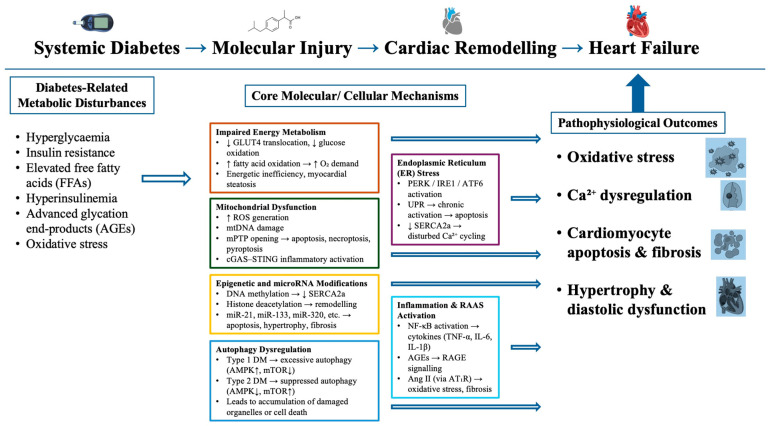
Integrated Molecular and Cellular Mechanisms of Heart Failure in Diabetes Mellitus: Schematic overview of the interconnected molecular and cellular mechanisms contributing to heart failure in diabetes mellitus. Chronic hyperglycemia, insulin resistance, and lipid overload activate mitochondrial dysfunction, endoplasmic reticulum stress, inflammation, dysregulated autophagy, and epigenetic reprogramming. These processes converge to induce oxidative stress, calcium mishandling, cardiomyocyte apoptosis, and fibrotic remodeling, culminating in diabetic heart failure.

The overall interplay between metabolic disturbances, molecular injury, and structural remodeling that culminates in diabetic HF is summarized in Figure 1.

## 2. Molecular and Cellular Mechanisms of Heart Failure in Diabetes Mellitus

### 2.1. Overview of Cardiac Energy Metabolism

Cardiac performance, both contraction and relaxation, relies heavily on efficient energy metabolism. The heart requires a constant and substantial supply of adenosine 5′-triphosphate (ATP) to sustain its contractile activity. Under physiological conditions, fatty acid (FA) oxidation within the mitochondria generates approximately 40–60% of the ATP needed by the myocardium, making FAs the principal energy source. Glucose serves as the second major substrate, contributing around 20–40% of ATP production through oxidative metabolism [10].

Circulating free fatty acids, derived either from serum albumin or from triacylglycerol (TAG) stored in lipoproteins, enter cardiomyocytes via fatty acid transporter proteins on the sarcolemma, most notably cluster of differentiation-36 (CD36) [11]. Once inside the cell, fatty acids can be utilized for the synthesis of lipid intermediates or transported into the mitochondrial matrix, where β-oxidation converts them into ATP [12]. Cardiac metabolic flexibility, the capacity to switch between substrates, is tightly regulated at several points in the FA oxidation pathway and influenced by factors such as changes in workload, hormonal fluctuations, oxygen availability, and the presence of alternative fuels like glucose [13,14]. Although fatty acids provide the bulk of the heart’s energy, they require more oxygen per ATP molecule produced, making them the least oxygen-efficient myocardial substrate. During physiological or pathological stress, mitochondrial FA oxidation can be suppressed, increasing reliance on glucose metabolism [15].

Glucose uptake into cardiomyocytes is mediated by both insulin-independent glucose transporter type 1 (GLUT-1) and insulin-dependent glucose transporter type 4 (GLUT-4), with GLUT-4 being the predominant isoform in the adult heart, accounting for roughly 70% of all cardiac glucose transporters. In contrast, GLUT-1 is more abundantly expressed in the fetal myocardium [16]. Insulin facilitates glucose transport by promoting the translocation of GLUT-4 from intracellular vesicles to the sarcolemmal membrane, and also by regulating GLUT gene expression [17]. Once inside the cell, glucose is phosphorylated to glucose-6-phosphate by hexokinase or converted to sorbitol via the polyol pathway. As illustrated in Figure 1, glucose-6-phosphate serves as a branching point for multiple metabolic pathways, including glycolysis, the hexose monophosphate (HMP) shunt, and the hexosamine biosynthetic pathway (HBP) [18].

### 2.2. Impaired Energy Metabolism in the Diabetic Heart

In DM, disturbances in both glucose and fatty acid metabolism play a pivotal role in the complex pathogenesis of HF [5]. A key defect in carbohydrate metabolism is the diminished expression and impaired translocation of glucose transporters, particularly GLUT-4, in cardiomyocytes. Experimental evidence from streptozotocin-induced type 1 diabetic rodent models has shown reduced GLUT-4 expression and defective trafficking in atrial tissue [19,20]. This alteration leads to decreased myocardial glucose uptake, glycolytic flux, and glucose oxidation, accompanied by a metabolic shift toward increased circulating free fatty acids (FFAs) and accelerated cardiac fatty acid oxidation [21].

The predominance of fatty acids as the primary myocardial energy substrate in DM represents a major metabolic hallmark. Elevated plasma FFAs and their enhanced oxidation not only suppress glycolysis and glucose oxidation but also increase mitochondrial oxygen demand. Since β-oxidation of FAs is less oxygen-efficient, this heightened oxygen consumption reduces overall cardiac energy conversion efficiency and depletes myocardial energetic reserves. Under conditions of increased workload, these constraints may precipitate functional decline and HF [21].

Beyond their role in ATP production, FFAs are utilized for the synthesis of triglycerides (TGs) and other lipid intermediates within cardiomyocytes. Excess reliance on FAs fosters excessive lipid accumulation in the myocardium, a phenomenon known as myocardial steatosis, which can exacerbate HF risk [21]. Such metabolic remodeling has been documented in both type 1 and type 2 DM, as well as in preclinical models [22,23].

The dual disruption of glucose metabolism and fatty acid utilization is not exclusive to DM and has been described in various cardiac pathologies, including HF [24]. Additionally, increased intracellular TGs and lipid intermediates impair insulin signaling, contributing to myocardial insulin resistance [25]. Cardiac insulin resistance, a defining feature of type 2 DM, serves as an independent risk factor for HF [26]. This impairment in insulin-mediated signaling arises from multiple interrelated mechanisms, including oxidative stress, chronic hyperglycemia, elevated lipid levels, and abnormal secretion of adipokines and cytokines [27].

### 2.3. Mitochondrial Dysfunction in Diabetes-Related Heart Failure

Mitochondria, the semi-autonomous organelles often referred to as the cell’s “powerhouses,” are essential for maintaining normal cellular physiology. They regulate ATP generation, calcium balance, oxidative stress responses, and apoptotic signaling [28]. In diabetes, mitochondrial dysfunction has emerged as a central contributor to the pathogenesis of HF [29]. In cardiomyocytes, approximately 90% of ATP is generated through oxidative phosphorylation within mitochondria [30].

As noted previously, diabetic hearts preferentially shift from glucose oxidation to fatty acid (FA) oxidation as the dominant ATP source [31]. While this adaptation sustains energy supply, it also impairs oxidative phosphorylation efficiency and enhances reactive oxygen species (ROS) production within the electron transport chain. Excess ROS inflicts oxidative damage on lipids and proteins, and, due to the mitochondrial genome’s proximity to the inner membrane, lack of histone protection, and limited repair capacity, mitochondrial DNA (mtDNA) is particularly susceptible [32].

**Figure 2:**
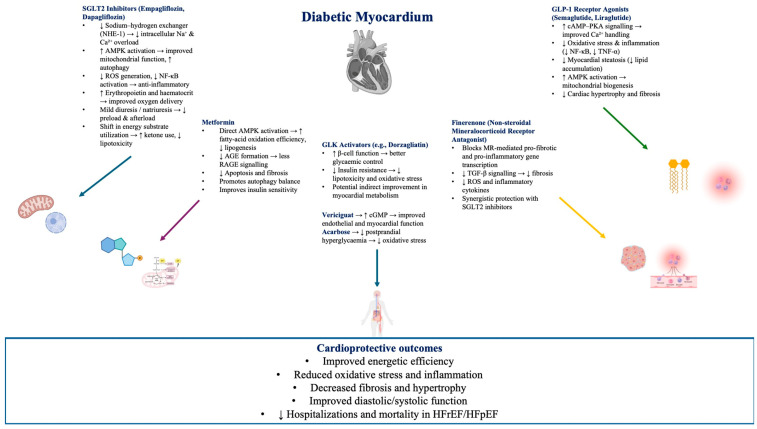
Molecular Targets and Cardioprotective Mechanisms of Antidiabetic Agents in Diabetes-Associated Heart Failure: Overview of the molecular targets and cardioprotective mechanisms of major antidiabetic drug classes in diabetes-associated heart failure. Sodium–glucose cotransporter 2 inhibitors, GLP-1 receptor agonists, metformin, and finerenone act through complementary pathways to improve cardiac metabolism, reduce oxidative stress and inflammation, restore calcium homeostasis, and limit fibrosis, collectively mitigating myocardial remodeling and heart-failure progression.

ROS also disrupt myocardial calcium (Ca^2+^) homeostasis, leading to cytosolic Ca^2+^ overload [33]. Elevated cytosolic Ca^2+^ can trigger the opening of the mitochondrial permeability transition pore (mPTP) [34]. As depicted in Figure 2, mPTP opening, along with mtDNA damage, initiates apoptotic cell death, driving mitochondrial impairment and progression toward HF [35]. Recent findings further indicate that increased mPTP activity can also induce necroptosis, a regulated form of necrotic cell death, in the diabetic myocardium [36].

Additionally, mtDNA damage may activate pyroptosis, a pro-inflammatory programmed cell death pathway, via stimulation of the cyclic GMP–AMP synthase–stimulator of interferon genes (cGAS–STING) axis [37]. This innate immune pathway detects cytosolic DNA, whether from pathogens or endogenous sources such as damaged mitochondria [38], and triggers inflammatory signaling. In cardiomyocytes, pyroptosis activation enhances inflammatory cascades and promotes myocardial hypertrophy, accelerating diabetic cardiomyopathy progression [39].

Overall, mitochondrial dysfunction in diabetic HF is mediated through multiple cell death modalities, including apoptosis, necroptosis, and pyroptosis. Other regulated death pathways, such as autophagic cell death, autosis, and ferroptosis, have also been identified in the diabetic heart and may contribute to disease progression [40].

### 2.4. Cardiac Endoplasmic Reticulum Stress (ERS) in Diabetes-Related Heart Failure

The endoplasmic reticulum (ER) is a key organelle responsible for multiple homeostatic functions, including lipid and steroid hormone biosynthesis, calcium regulation, post-translational modification of secretory and membrane proteins, and the folding of nascent polypeptides [41]. Endoplasmic reticulum stress (ERS) occurs when ER homeostasis is persistently disrupted, resulting in the accumulation of misfolded or unfolded proteins. This can be triggered by various factors such as impaired glycosylation, nutrient deprivation, oxidative stress, and other metabolic insults. In response, cells activate the unfolded protein response (UPR), a coordinated signaling network aimed at restoring ER balance [42].

Under physiological conditions, the UPR reduces global protein synthesis, enhances the production of ER-resident chaperones, and promotes degradation of misfolded proteins, thereby re-establishing proteostasis [43]. Three main ER stress sensors—protein kinase R-like ER kinase (PERK), inositol-requiring enzyme-1 (IRE1), and activating transcription factor-6 (ATF6)—initiate this pathway when released from inhibitory binding to ER chaperones [44]. Upon activation, these sensors induce transcription of UPR target genes, downregulate protein translation, and upregulate proteins involved in ER-associated degradation [45].

Although the UPR is initially protective, chronic or excessive ER stress can activate ER-mediated apoptotic pathways in cardiomyocytes, contributing to contractile dysfunction [46]. Persistent ERS is increasingly recognized as a feature of metabolic disorders, including obesity, diabetes, and age-related cardiovascular disease [47].

In diabetes mellitus (DM), hyperglycemia, elevated circulating free fatty acids (FFAs), inflammation, metabolic shifts, and lipotoxicity converge to promote ER stress in the heart [48,49]. In turn, activated ERS triggers UPR signaling, leading to cardiomyocyte apoptosis, a phenomenon confirmed in diabetic rodent models through upregulation of ER stress markers and pro-apoptotic proteins [50]

One proposed mechanism linking ERS to diabetic HF involves dysregulation of sarcoplasmic reticulum Ca^2+^-ATPase (SERCA). SERCA, an ATP-dependent membrane pump, sequesters Ca^2+^ into the sarcoplasmic reticulum to support normal excitation–contraction coupling. In diabetes, hyperglycemia-induced non-enzymatic glycation may impair SERCA structure and function [51]. Reduced SERCA activity disrupts calcium cycling, promotes diastolic dysfunction, and impairs contractility, thereby increasing the risk of HF [52,53,54].

Moreover, ER stress has been shown to alter myocardial energetics, affecting substrate utilization, oxidative phosphorylation efficiency, and ATP delivery to the contractile apparatus [55]. Collectively, these disturbances can lead to left ventricular hypertrophy, cardiomyopathy, and a decline in cardiac output, ultimately progressing to HF [56].

Although the precise molecular events linking ER stress to diabetic cardiomyopathy require further investigation, targeting ERS and its downstream pathways may represent a promising therapeutic approach for preventing or mitigating diabetes-associated HF.

### 2.5. Role of Inflammation in Diabetes-Related Heart Failure

Systemic, low-grade chronic inflammation is a hallmark of diabetes mellitus (DM) and plays a pivotal role in the pathogenesis of both microvascular and macrovascular complications [57]. In the context of the heart, persistent inflammatory signaling, driven by hyperglycemia, hyperlipidemia, hyperinsulinemia, and insulin resistance, contributes significantly to the onset and progression of diabetic HF [58].

Several interlinked molecular mechanisms connect metabolic derangements in DM to cardiac inflammation. Sustained hyperglycemia promotes oxidative stress and the accumulation of advanced glycation end products (AGEs), while elevated lipid levels, especially oxidized low-density lipoprotein (oxLDL), further exacerbate tissue injury. These metabolic insults converge on the nuclear factor kappa-B (NF-κB) pathway, the renin–angiotensin–aldosterone system (RAAS), and excessive production of pro-inflammatory interleukins, collectively driving myocardial remodeling and dysfunction [59,60].

### 2.6. NF-κB–Mediated Inflammatory Cascade

Prolonged hyperglycemia disrupts cardiac glucose metabolism, suppressing glucose oxidation and favoring fatty acid utilization. Enhanced fatty acid oxidation elevates lipoprotein levels, particularly oxLDL, which is known to activate NF-κB signaling [61,62]. Normally sequestered in the cytoplasm in an inactive form, NF-κB becomes activated under these conditions, translocates to the nucleus, and binds to specific DNA sequences to initiate transcription of pro-inflammatory cytokines, chemokines, adhesion molecules, transforming growth factor-β (TGF-β), pro-apoptotic genes, and other stress-response proteins [63]. The cumulative effect of this signaling includes oxidative injury to the myocardium, interstitial fibrosis, hypertrophy, cardiomyocyte death, and early diastolic dysfunction—key pathological features of diabetic HF [64]. Given its central role in inflammation, NF-κB is regarded as a promising therapeutic target for preventing chronic diabetes-related complications, including HF [4].

### 2.7. RAAS Activation in the Diabetic Heart

DM can also upregulate RAAS activity within the myocardium via multiple mechanisms:

1. Direct hyperglycemia-induced Angiotensin II (Ang-II) synthesis—Elevated glucose levels stimulate cardiomyocyte production of Ang-II, largely through chymase-dependent pathways. Chymases, a family of serine proteases expressed in mast cells, fibroblasts, and vascular endothelial cells, facilitate intracellular Ang-II formation, which has been linked to apoptosis, oxidative stress, and myocardial fibrosis in diabetic models [65,66]. Chymase inhibition has shown notable therapeutic potential in halting cardiac and vascular injury in DM [67,68].
2. Increased myocardial sensitivity to Ang-II—Hyperglycemia enhances tissue responsiveness to Ang-II, promoting more pronounced vasoconstriction and contractile responses. In experimental models, elevated glucose augmented Ang-II–mediated aortic contraction through activation of the angiotensin type-1 receptor (AT1R) [69].
3. AGE-mediated RAAS stimulation—AGEs, formed under conditions of sustained hyperglycemia, oxidative stress, and dyslipidemia, can activate intracellular signaling cascades that promote pro-inflammatory mediator release, oxidative stress, and RAAS activation. Importantly, AGEs also potentiate chymase-dependent Ang-II production [70,71,72].
4. Ang-II exerts multiple effects that initially serve as compensatory mechanisms, such as vasoconstriction, stimulation of growth factors, and promotion of vascular smooth muscle cell and fibroblast proliferation, but over time, these changes contribute to maladaptive remodeling, hypertrophy, and progression to HF [73].

### 2.8. Role of Autophagy in Diabetes-Related Heart Failure

Autophagy refers to the lysosome-mediated degradation and recycling of intracellular components, a fundamental process in all cell types, including cardiomyocytes, for maintaining homeostasis and adapting to nutritional, metabolic, or infection-related stress [74]. In mammalian cells, three main forms of autophagy are recognized, classified by the mode of cargo delivery to the lysosome: microautophagy, macroautophagy, and chaperone-mediated autophagy (CMA) [75].

• Microautophagy involves direct invagination of the lysosomal or vacuolar membrane to engulf cytoplasmic material.
• CMA selectively targets soluble cytosolic proteins for lysosomal degradation via chaperone proteins.
• Macroautophagy (hereafter “autophagy”)—the most extensively studied sequesters cellular cargo within double-membraned autophagosomes, which subsequently fuse with lysosomes for breakdown and recycling [76,77].

More than 30 autophagy-related (ATG) genes and proteins orchestrate this process, governing initiation, cargo selection, vesicle formation, fusion, and degradation [78]. Among the regulatory pathways, the mammalian target of rapamycin (mTOR) serves as the primary inhibitor, while AMP-activated protein kinase (AMPK) acts as a major activator [79].

• mTOR, a conserved serine/threonine kinase, inhibits autophagosome initiation by phosphorylating ATG13 and ULK1/2 (the mammalian homologs of yeast ATG1), disrupting ULK complex function and blocking downstream autophagy activation [80,81].
• AMPK, an intracellular energy sensor, stimulates autophagy both directly, by phosphorylating ULK1 and other ATG proteins, and indirectly, by regulating autophagy-related gene expression via transcription factors [82].

In a healthy heart, autophagy maintains protein quality control and prevents accumulation of dysfunctional organelles and toxic aggregates. However, evidence suggests that autophagic flux is frequently dysregulated in the diabetic myocardium [83]. Findings are inconsistent regarding whether autophagy is upregulated or suppressed, and patterns may differ between type 1 and type 2 DM.

#### 2.8.1. Autophagy in Type 1 Diabetes Mellitus

Some experimental models of type 1 DM show enhanced autophagy flux, attributed to insulin deficiency driven impairment of GLUT4 and FAT/CD36 translocation, which limits glucose and fatty acid uptake and mimics a nutrient-deprivation state. This energy shortage activates AMPK, thereby promoting autophagy [84,85]. While upregulated autophagy has been associated with diastolic dysfunction in diabetic cardiomyopathy [86], other studies suggest that reduced autophagy may be an adaptive response to protect against contractile impairment [87,88].

#### 2.8.2. Autophagy in Type 2 Diabetes Mellitus

Conversely, type 2 DM is often characterized by suppressed autophagy. Chronic nutrient excess, obesity, and hyperlipidemia elevate intracellular energy substrates, reducing AMPK activation and thereby downregulating autophagy [89]. This suppression limits glucose transport, glycolysis, fatty acid oxidation, and overall ATP generation, contributing to energy deficit and contractile dysfunction. Indeed, reduced AMPK activity is recognized as a key driver of diabetic HF in type 2 DM [84].

Although impaired autophagy and reduced cardiomyocyte viability are linked to functional decline in the diabetic heart [70] contradictory evidence exists, with some reports indicating that autophagy suppression may in certain contexts protect against myocardial injury and HF [90,91].

The exact role of autophagy in diabetic HF—whether protective, detrimental, or context-dependent—remains unresolved. Further mechanistic studies are required to clarify whether therapeutic modulation of autophagy should aim to enhance or suppress this pathway in type 1 versus type 2 DM.

## 3. Role of Epigenetics in Diabetes-Related Heart Failure

Epigenetics refer to heritable modifications in gene expression that occur without altering the underlying DNA sequence. The two principal epigenetic mechanisms are DNA methylation and histone modification, both of which regulate gene transcription and have been implicated in the pathogenesis of diabetes mellitus (DM) and its cardiovascular complications [92,93,94].

### 3.1. DNA Methylation

DNA methylation occurs when DNA methyltransferases (DNMTs) add methyl groups to cytosine or adenine bases, most commonly at the 5′ position of cytosine residues within CpG dinucleotides. This modification generally suppresses gene expression by blocking transcription factor binding [95].

Emerging evidence links aberrant DNA methylation to diabetic complications, including HF [96]. In a hyperglycemic environment, tumor necrosis factor-α (TNF-α) can enhance DNMT activity, leading to hypermethylation of the SERCA promoter in cardiomyocytes [97]. This reduces SERCA protein expression, impairing calcium handling and contributing to diastolic dysfunction and reduced contractility hallmarks of diabetic HF [98]. Elevated TNF-α levels have also been correlated with HF severity [99]. Moreover, abnormal DNA methylation in human cardiomyocytes has been associated with contractile impairment, mitochondrial dysfunction, and disturbances in lipid and glucose metabolism. Collectively, these findings suggest that DNA methylation–driven adverse remodeling plays a role in the progression of diabetic HF, although further mechanistic studies are warranted.

### 3.2. Histone Modifications

Histones, H2A, H2B, H3, and H4, combine with DNA to form nucleosomes, the fundamental units of chromatin. Post-translational modifications of histones, including methylation, acetylation, phosphorylation, ubiquitination, ADP-ribosylation, and adenylation, influence chromatin structure and gene transcription [100,101]. These changes can alter histone–protein interactions, modify local hydrophobicity, affect RNA polymerase activity, and modulate the recruitment of transcriptional coactivators [102].

In the diabetic myocardium, hyperglycemia-induced mitochondrial reactive oxygen species (ROS) production suppresses JunD, a transcription factor that acts as a protective gatekeeper against oxidative stress. Loss of JunD function has been linked to cardiac dysfunction in both experimental and clinical settings [103]. ROS also promote sustained epigenetic activation of the NF-κB p65 subunit in vascular cells, partly through histone H3 lysine 4 methylation, which enhances pro-inflammatory gene expression [104].

Additionally, dysregulation of the balance between histone acetylation and deacetylation has been implicated in diabetic HF, with evidence suggesting that excessive histone deacetylation may contribute to pathological cardiac remodeling [105].

Epigenetic modifications—through both DNA methylation and histone changes—emerge as critical molecular mediators of diabetes-related HF. These processes influence inflammatory signaling, oxidative stress responses, calcium handling, and metabolic regulation. Given their reversible nature, epigenetic targets represent a promising avenue for future cardiovascular therapeutics in the context of DM [106].

### 3.3. Role of MicroRNAs in Diabetes-Related Heart Failure

MicroRNAs (miRNAs) are small, endogenous, noncoding RNA molecules, approximately 22 nucleotides in length, that regulate gene expression by either suppressing mRNA translation or promoting mRNA degradation [107]. In the myocardium of individuals with diabetes mellitus (DM), miRNA expression profiles differ significantly from those in healthy controls [108]. Alterations in miRNA synthesis and abundance have been implicated in cardiac remodeling and the pathogenesis of diabetic HF [109].

Dysregulated miRNAs in DM can modulate key pathological pathways, including mitochondrial dysfunction, reactive oxygen species (ROS) production, calcium homeostasis disturbances, apoptosis, and fibrosis, all of which contribute to cardiomyocyte hypertrophy, maladaptive remodeling, and HF progression. Cardiomyocyte hypertrophy, a hallmark structural change in early HF, has been linked to aberrant miRNA expression in diabetic hearts [110].

One example is miR-133, which is abundantly expressed in cardiac tissue and plays a crucial role in diabetic cardiac hypertrophy [111]. In a clinical study involving type 2 DM patients with diabetic cardiomyopathy, miR-17 was found to be upregulated, while miR-24, miR-150, miR-199a, miR-214, and miR-320a were downregulated compared to healthy subjects. This pattern suggested that circulating miRNA dysregulation may contribute to disease pathogenesis.

Furthermore, nuclear miR-320 upregulation has been shown to worsen diabetic cardiac dysfunction by activating the transcription of fatty acid metabolism genes, leading to lipotoxicity in the myocardium [112]. Based on such findings, combining circulating miRNA profiling with conventional biomarkers has been proposed as a strategy to improve the early diagnosis of diabetic cardiomyopathy [113].

In experimental diabetic mouse models, miR-21 levels were markedly reduced in hearts exhibiting diastolic dysfunction—an early feature of HF. Restoring miR-21 expression protected against diastolic dysfunction by targeting gelsolin, a regulator of actin filament dynamics [114].

Importantly, miRNAs may also interact with other epigenetic mechanisms, influencing histone H2A mRNA levels, histone deacetylase activity, and DNA methyltransferase expression, thereby modifying chromatin structure and gene transcription [115]. Despite these advances, the precise roles of specific miRNAs in diabetic HF, and their interplay with broader epigenetic processes, remain incompletely understood.

## 4. Pathomechanisms in Diabetes-Associated Heart Attacks

Diabetes profoundly alters myocardial biology, rendering the heart intrinsically more vulnerable to ischemic injury beyond the effects of atherosclerosis. The diabetic myocardium exhibits metabolic inflexibility, shifting toward excessive fatty acid oxidation and diminished glucose utilization, which elevates oxygen demand, drives oxidative stress, and impairs ATP production. Mitochondrial dysfunction, characterized by reactive oxygen species accumulation, mitochondrial DNA damage, and impaired mitophagy, further compromises cellular energetics. Parallel stress responses, including maladaptive endoplasmic reticulum stress and dysregulated autophagy, disrupt protein quality control and promote cardiomyocyte death. Emerging evidence also implicates regulated necrosis forms such as necroptosis and pyroptosis, which couple metabolic injury to inflammatory amplification through RIPK1/RIPK3 and inflammasome–gasdermin pathways. Chronic activation of NF-κB, NLRP3, and renin–angiotensin–aldosterone signaling sustains a proinflammatory, profibrotic milieu, while aldosterone-mediated oxidative stress and impaired nitric oxide signaling aggravate remodeling. On a regulatory level, epigenetic alterations, including DNA methylation, histone modifications, and microRNA dysregulation, lock in maladaptive transcriptional programs that perpetuate injury even after glycemic normalization. Collectively, these interwoven metabolic, inflammatory, neurohormonal, and epigenetic perturbations create a self-reinforcing cycle of oxidative stress, fibrosis, and cardiomyocyte loss, explaining the greater infarct size, poorer recovery, and higher mortality observed in diabetic myocardial infarction.

## 5. Pharmacological Management of Heart Failure

Before 2010, a substantial number of randomized controlled trials (RCTs) had been completed in chronic HF, leading to the establishment of the traditional “golden triangle” of therapy: angiotensin-converting enzyme inhibitors (ACEIs) or angiotensin receptor blockers (ARBs), β-blockers, and mineralocorticoid receptor antagonists (MRAs). Since 2019, numerous RCTs have investigated novel therapeutic agents for HF, including angiotensin receptor–neprilysin inhibitors (ARNIs), sodium–glucose cotransporter 2 (SGLT2) inhibitors, and ivabradine. The PARADIGM-HF trial demonstrated that ARNIs, particularly sacubitril/valsartan, should be prioritized for patients with HF with reduced ejection fraction (HFrEF) [116].

Vericiguat, a soluble guanylate cyclase (sGC) stimulator, gained approval in several countries between 2021 and 2022 for HFrEF, showing benefit in reducing the risk of hospitalization for HF or the need for urgent intravenous diuretics [117]. Finerenone, a third-generation MRA with enhanced renal and cardiac protective effects, received its first approval in the United States in 2021 and has since been endorsed by multiple national and international guidelines [118].

By 2021, the therapeutic paradigm had evolved into a “new quadruple” regimen consisting of ARNI (or ACEI/ARB), SGLT2 inhibitor, β-blocker, and MRA. Early initiation of this combination has been shown to yield sustained benefits, including a 62% reduction in cardiovascular mortality or HHF risk. Despite this progress, most of the evidence supporting these agents, particularly ARNIs and SGLT2 inhibitors, originates from HFrEF populations, although both have also shown the capacity to reduce HHF in HF with preserved ejection fraction (HFpEF).

Certain patient groups, such as those with low blood pressure or tachycardia, face limitations in using therapies like ARNIs and β-blockers. In contrast, SGLT2 inhibitors have fewer hemodynamic constraints, are generally well tolerated, and have a favorable safety profile. Large-scale clinical trials are ongoing to further explore their role in HF management [119], although an intervention that significantly extends survival in HFpEF has yet to be identified.

### 5.1. Antidiabetic Agents and Heart Failure

DM is a well-established independent risk factor for HF, and ideally, effective management of one condition should positively impact the other without causing harm (Table 1). However, certain glucose-lowering agents either have no significant effect on HF or may worsen outcomes. Table 2 summarizes HF-relevant effects and clinical recommendations across glucose-lowering drug classes, highlighting benefits with SGLT2 inhibitors/GLP-1 RAs, metformin’s favorable profile in stable HF, and the need for caution or avoidance with sulfonylureas, insulin, and TZDs.

Traditional thiazolidinediones (TZDs), which primarily activate peroxisome proliferator-activated receptor-γ (PPARγ), can lead to excessive water and sodium retention when overstimulated [120]. Large clinical trials such as the RECORD and PROactive studies reported that TZDs significantly increased the risk of HF and mortality [121]. In contrast, chiglitazar sodium, a full PPAR agonist, has not been associated with HF in trials and shows lower rates of edema, fractures, and weight gain [122]. A meta-analysis of 115 studies found sulfonylureas to be linked with higher mortality [123], while retrospective analyses suggest insulin use in HF patients with type 2 DM (T2DM) correlates with worse outcomes, though randomized controlled trials (RCTs) have not confirmed this association [124]. Given these concerns, both sulfonylureas and insulin are generally reserved as second- or third-line agents in T2DM patients with HF, though their safety remains debated [125].

Some agents demonstrate potential benefit. The MeRIA trial found that acarbose reduced HF risk by up to 45% in T2DM patients [126]. However, the 2013 SAVOR-TIMI 53 trial reported that saxagliptin, a dipeptidyl peptidase-4 (DPP-4) inhibitor, was non-inferior to placebo in cardiovascular safety but increased hospitalization for HF [127]. Similarly, the EXAMINE trial found no significant differences in cardiovascular outcomes between alogliptin and placebo [128]. Data from meta-analyses and preclinical studies on DPP-4 inhibitors remain inconsistent.

Metformin, long avoided in HF due to concerns over lactic acidosis [129], is now recognized for its cardioprotective properties. It activates AMP-activated protein kinase (AMPK), reduces cardiomyocyte apoptosis, limits advanced glycation end product (AGE) formation, enhances mitochondrial β-oxidation of fatty acids, and improves myocardial performance [130]. Metformin may also modulate gut microbiota composition, particularly increasing short-chain fatty acid-producing bacteria in HF patients [131]. Systematic reviews have shown that metformin lowers all-cause and cardiovascular mortality and reduces HF readmission risk in T2DM patients [132]. Since 2007, the American Diabetes Association has recommended metformin for HF patients with stable renal function [133].

Other novel therapies include glucokinase activators (GKAs) such as dorzagliatin, which fully activate glucokinase by stabilizing its active conformation, thereby improving β-cell performance, reducing insulin resistance, and restoring glycemic control in T2DM [134]. In a phase III extension trial, dorzagliatin increased the time in target glucose range (TIR) from 59.9% at baseline to 83.7% after 46 weeks of treatment [135]. Since each 10% rise in TIR has been linked to a 6% reduction in major adverse cardiovascular events (MACE), dorzagliatin’s cardiovascular benefits warrant further prospective evaluation [136].

Glucagon-like peptide-1 receptor agonists (GLP-1 RAs) may improve cardiovascular health by reducing appetite and weight through central nervous system effects, decreasing hepatic steatosis, and lowering triglyceride and LDL-C levels [137]. In HFpEF, they reduce atrial enlargement and epicardial fat; in HFrEF, they attenuate adverse ventricular remodeling, suppress cardiac inflammation, and enhance AMPK activity. Notably, the STEP-HFpEF and STEP-HFpEF DM trials showed that semaglutide significantly improved HF-related symptoms and reduced body weight in obesity-related HFpEF [138].

Sodium–glucose cotransporter 2 (SGLT2) inhibitors have demonstrated efficacy in lowering HbA1c, body mass index (BMI), and systolic blood pressure, alongside reducing HHF risk [139]. However, concerns persist regarding increased genitourinary (GU) infections, especially urinary tract infections, which may limit broader uptake [140].

The Grading of Recommendations Assessment, Development, and Evaluation (GRADE) approach, adopted by over 110 organizations worldwide, assesses evidence quality using five downgrading factors: risk of bias, inconsistency, imprecision, indirectness, and publication bias [141]. According to GRADE evaluations, SGLT2 inhibitors are strongly recommended in major guidelines with high-quality evidence (Grade A) for both HFrEF and HFpEF [142].

### 5.2. SGLT2 Inhibitors and Heart Failure

Multiple landmark clinical trials have confirmed the efficacy of sodium–glucose cotransporter 2 (SGLT2) inhibitors in HF through rigorous prospective evaluations. In HFrEF, the EMPEROR-Reduced and DAPA-HF trials demonstrated that these agents significantly lower the risk of hospitalization for HF, reduce cardiovascular mortality, and slow the progression of chronic kidney disease (CKD) [143]. For HFpEF, the EMPEROR-Preserved and DELIVER trials revealed that SGLT2 inhibitors not only reduced the combined endpoint of cardiovascular death and HHF but also modestly improved patient-reported symptoms, as measured by the Kansas City Cardiomyopathy Questionnaire (KCCQ) [144]. Evidence from large real-world database analyses has further validated these cardiovascular benefits. Table 1 collates key designs and headline outcomes from pivotal randomized trials and large real-world datasets evaluating SGLT2 inhibitors in HF.

#### Potential Mechanisms of Action for SGLT2 in HF

While SGLT2 inhibitors lower plasma glucose levels by promoting urinary glucose excretion, their cardiovascular benefits appear largely independent of their glucose-lowering effects [145]. Sustained improvements in cardiovascular outcomes may stem from multiple synergistic mechanisms, including mild diuresis, enhanced natriuresis, favorable ventricular remodeling, improved cardiac function, inhibition of sodium–hydrogen exchangers, optimization of myocardial energy metabolism, suppression of sympathetic overactivity, and anti-inflammatory and antioxidant effects [146]. Figure 2 illustrates how current pharmacologic agents modulate these interconnected molecular pathways to confer cardioprotective effects in diabetes-associated HF.

Data from the EMPA-REG OUTCOME trial indicated that nearly half of the cardiovascular benefit could be attributed to increases in hematocrit. Interestingly, in T2DM patients treated with dapagliflozin, erythropoietin (EPO) levels remained elevated even after two months, suggesting that hematocrit increases result from both diuretic-induced hemoconcentration and enhanced EPO production [147].

Additional vascular benefits may be mediated through improved endothelial function via increased nitric oxide (NO) bioavailability and reduced NO degradation [148]. SGLT2 inhibitors may also enhance cardiac performance by reducing epicardial fat mass and promoting more efficient fuel utilization, shifting myocardial metabolism away from glucose dependence toward fatty acids and ketones, which require less oxygen [149]. Dapagliflozin, for instance, directly inhibits the sodium–hydrogen exchanger (NHE-1), lowering intracellular sodium concentrations and improving ionic homeostasis.

Recent studies suggest these drugs may also exert antioxidant effects by upregulating heme oxygenase, decreasing advanced glycation end products (AGEs), and leveraging the antioxidative potential of ketone metabolism [150]. Anti-inflammatory pathways may involve reduced nuclear factor-κB (NF-κB) activation and diminished production of pro-inflammatory cytokines and chemokines [151]. Additionally, SGLT2 inhibitors have been shown to significantly increase ketone body concentrations, particularly in diabetic individuals, potentially inhibiting NLRP3 inflammasome activation [152].

Other contributing benefits include weight loss, improved insulin sensitivity, and reductions in serum uric acid levels [153].

In summary, while the clinical benefits of SGLT2 inhibitors in HF are well established, a deeper understanding of their multifaceted mechanisms could not only clarify their therapeutic impact but also provide broader insights into HF pathophysiology and new treatment opportunities.

### 5.3. Finerenone—Non-Steroidal Mineralocorticoid Receptor Antagonists in Heart Failure

Mineralocorticoid receptor (MR) activation, even in the absence of aldosterone, can drive the production of reactive oxygen species, trigger inflammatory pathways, and promote fibrosis. These processes contribute to myocardial hypertrophy, adverse ventricular remodeling, glomerular hypertrophy, and glomerulosclerosis, ultimately worsening both cardiac and renal outcomes [154]. This has made targeted mineralocorticoid receptor antagonism a key focus of recent drug development.

Finerenone, the first novel non-steroidal MRA approved worldwide for type 2 DM (T2D)-associated chronic kidney disease (CKD) [155], exerts its effects by interacting with multiple MR sites in the glomeruli, renal tubules, and myocardium. It binds selectively to Ala-773 and Ser-810 residues, inducing C-terminal ligand-dependent interactions. This conformational change causes helix 12 in the AF2 activation domain to protrude, locking the receptor into an “inactive conformation” and completely blocking co-regulator recruitment [156].

Unlike steroidal MRAs, finerenone shows no affinity for glucocorticoid, androgen, or progesterone receptors, which enhances its selectivity and confers direct anti-inflammatory and anti-fibrotic actions [157]. Its pharmacokinetic profile, characterized by the inability to cross the blood–brain barrier, a short half-life, and the potential for faster correction of hyperkalemia, supports both efficacy and safety.

Two pivotal phase III trials, FIDELIO-DKD and FIGARO-DKD, demonstrated that finerenone, when added to the maximum tolerated dose of a renin–angiotensin–aldosterone system (RAAS) inhibitor, significantly reduced proteinuria, cardiovascular events, and renal disease progression [158]. In recognition of these benefits, the European Society of Cardiology (ESC) has given finerenone its highest-level recommendation for cardiovascular disease management in patients with both acute and chronic HF and DM [159].

A pooled analysis from the FIDELITY study indicated that the cardiovascular and renal benefits of finerenone were consistent regardless of concurrent SGLT2 inhibitor therapy [155]. However, patients receiving both agents experienced greater combined renal–cardiac protection and a lower incidence of hyperkalemia compared to placebo (10.3% vs. 2.7%) [160].

Since both SGLT2 inhibitors and finerenone can cause a temporary reduction in estimated glomerular filtration rate (eGFR), sequential rather than simultaneous initiation is advised to optimize safety. The 2023 China Expert Consensus recommends starting finerenone at least 2–4 weeks after SGLT2 inhibitor initiation and closely monitoring eGFR changes throughout treatment.

### 5.4. Vericiguat—An Emerging Therapy in Heart Failure Management

The 2021 ESC Heart Failure Guidelines incorporated, for the first time, the next-generation soluble guanylate cyclase (sGC) stimulator, vericiguat [161]. sGC is an essential enzyme responsible for generating cyclic guanosine monophosphate (cGMP) upon binding with nitric oxide (NO), a process critical for regulating vascular tone, cardiac contractility, and structural remodeling of the heart.

Vericiguat works through a dual mechanism:

1. NO-dependent pathway—it enhances the sensitivity of sGC receptors to NO by stabilizing the NO-sGC binding site, leading to increased cGMP synthesis in cardiomyocytes and vascular smooth muscle cells.
2. NO-independent pathway—it binds directly to an alternative site on the sGC receptor, further boosting cGMP production [117].

By restoring the NO–sGC–cGMP signaling cascade, vericiguat improves endothelial and vascular function, reduces ventricular remodeling, and limits myocardial hypertrophy.

The VICTORIA phase III trial demonstrated that vericiguat lowered the annual absolute risk (ARR) of the primary composite endpoint by 4.2%, with a number needed to treat (NNT) of 24, meaning that treating 24 patients for one year prevents one primary endpoint event [162]. Long-term therapy with vericiguat was also associated with improvements in left ventricular ejection fraction (LVEF), reductions in NT-proBNP levels, and enhanced patient-reported quality of life [163]. Importantly, the treatment showed no negative effects on blood pressure or renal function, underscoring its safety profile [164].

For patients with worsening HFrEF, despite optimal quadruple therapy, there remains a substantial residual risk of hospitalization and adverse events [165]. Given the VICTORIA trial results, regulatory approval was granted for vericiguat in the treatment of symptomatic chronic HF with an ejection fraction below 45% following a recent worsening event, due to its favorable tolerability, safety, and its ability to reduce the rate of hospitalization for HF (HHF) [166].

### 5.5. Considerations in Selecting Research Endpoints for SGLT2 Inhibitor Trials in HFpEF

Choosing appropriate clinical trial endpoints for novel drug evaluations remains a significant challenge. Since the 1960s, randomized clinical trials have relied on predefined primary endpoints, often outlined in a non-stratified fashion within the study protocol. However, specifying multiple primary endpoints without adequately addressing multiplicity can lead to false-positive conclusions, where a treatment appears effective simply because one endpoint meets the conventional *p*-value threshold of 0.05 [167].

A notable example is the EMPEROR-Preserved trial, which assessed SGLT2 inhibitors in HFpEF patients using a composite endpoint of cardiovascular death or hospitalization for HF. While the trial was the first large-scale HFpEF study to achieve its primary endpoint, the observed benefit was largely driven by reductions in HF. There were no statistically significant improvements in cardiovascular or all-cause mortality, quality-of-life scores, or NT-proBNP levels [167].

## 6. Comprehensive Discussion and Future Perspectives

Diabetes-associated HF is increasingly recognized as a distinct clinical and pathophysiological entity, driven by a network of interconnected metabolic, molecular, and structural alterations. As our understanding of these mechanisms deepens, opportunities are emerging to develop targeted interventions that address the root causes of myocardial dysfunction in DM.

Several therapeutic directions are particularly promising. First, metabolic modulation, specifically enhancing glucose oxidation while limiting excessive fatty acid utilization, could improve cardiac efficiency and reduce lipotoxic injury. Agents that shift substrate preference, such as trimetazidine or ranolazine, alongside sodium–glucose cotransporter 2 (SGLT2) inhibitors, warrant further evaluation in diabetic HF cohorts.

Mitochondrial protection is another critical area. Strategies to restore mitochondrial dynamics, reduce oxidative stress, and improve ATP generation may halt or reverse myocardial injury. This includes targeting mitochondrial permeability transition pore (mPTP) opening, enhancing mitophagy, and modulating ROS scavenging pathways.

Addressing endoplasmic reticulum stress (ERS) through chemical chaperones or modulators of the unfolded protein response (UPR) could help preserve protein quality control and calcium handling in diabetic cardiomyocytes. Similarly, anti-inflammatory interventions, whether by blocking NF-κB activation, modulating the renin–angiotensin–aldosterone system (RAAS), or reducing advanced glycation end-product (AGE) signaling, have potential to attenuate myocardial remodeling and fibrosis.

Epigenetic modifications and microRNAs (miRNAs) offer emerging avenues for precision medicine. Because these changes are reversible, they present the possibility of fine-tuning gene expression involved in calcium cycling, oxidative stress, and fibrotic pathways. Therapeutics based on miRNA mimics, antagomirs, or epigenetic enzyme inhibitors could complement conventional treatment.

Finally, autophagy modulation remains an area of active debate. The context-dependent nature of autophagy, protective in some stages of disease and detrimental in others, requires a more nuanced understanding to guide its therapeutic targeting.

Many randomized controlled trials (RCTs) predominantly enroll patients with HFrEF to maximize statistical power, but this approach often overlooks the broad heterogeneity of HF phenotypes [168]. Moving forward, research should prioritize studies that stratify participants based on distinct HF subtypes, shifting from large, mixed populations to more targeted mechanistic investigations in smaller, homogeneous cohorts [169].

Evidence suggests that patients with both DM and/or HF may particularly benefit from SGLT2 inhibitor therapy, warranting further exploration of drug synergies [137]. For example, recent cohort data indicate that combining a GLP-1 receptor agonist (GLP-1 RA) with an SGLT2 inhibitor reduces the risk of MACE and serious renal complications compared to using either agent alone [137]. The potential cardiovascular and renal advantages of pairing SGLT2 inhibitors with mineralocorticoid receptor antagonists (MRAs) are currently being evaluated in the MIRACLE and CONFIDENCE trials [170].

Beyond symptomatic HF management, SGLT2 inhibitors are also being studied for prevention strategies targeting upstream disease mechanisms. Given the strong association between atrial fibrillation (AF) and HFpEF, it is notable that SGLT2 inhibitors have been linked to reduced AF incidence in post hoc analyses of RCT adverse event data [171]. Furthermore, initiating SGLT2 inhibitors in patients with T2DM and a history of percutaneous coronary intervention (PCI) was associated with lower rates of adverse cardiorenal outcomes and mortality, independent of the interval since PCI [172].

Finally, ongoing large-scale trials, DAPA-MI and EMPACT-MI, are assessing whether early initiation of SGLT2 inhibitors after myocardial infarction can improve cardiovascular outcomes [142].

Future research must prioritize comprehensive translational studies that validate key targets across diverse cohorts and define the temporal evolution of these pathomechanisms during different stages of DM and acute ischemic injury. Integrating multi-omics approaches, combining genomics, transcriptomics, proteomics, and metabolomics, can illuminate novel networks and therapeutic nodes. Additionally, advancing relevant preclinical models that faithfully recapitulate diabetic myocardial injury will accelerate the bench-to-bedside pipeline.

## 7. Conclusions

Diabetic HF is the culmination of a complex web of interrelated mechanisms, ranging from impaired energy metabolism and mitochondrial dysfunction to ER stress, chronic inflammation, dysregulated autophagy, epigenetic alterations, and miRNA dysregulation. Each of these pathways not only contributes independently to myocardial injury but also interacts with others, creating a self-perpetuating cycle of structural and functional decline.

Current evidence suggests that targeting upstream drivers, such as metabolic inflexibility, oxidative stress, calcium mishandling, and inflammatory activation, could substantially modify disease progression. In parallel, modulating reversible regulators like epigenetic marks and miRNAs offers a route toward highly personalized therapies. Novel therapeutic modalities aimed at restoring mitochondrial homeostasis, inhibiting inflammasome activation, regulating programmed necrotic cell death, and reversing detrimental epigenetic changes show encouraging potential but remain largely at the preclinical stage. Future research priorities should include translational and clinical studies that validate these targets in diverse patient populations, alongside implementation of precision medicine frameworks to stratify patients by molecular phenotype. Delineating the temporal evolution of these pathomechanisms during diabetes progression and acute ischemic events will further enable optimization of treatment timing and target selection.

However, significant challenges remain. The relative contributions of these mechanisms likely vary between patients, between type 1 and type 2 DM, and across disease stages. Moreover, most mechanistic insights stem from preclinical or small-scale clinical studies, underscoring the urgent need for translational research and randomized controlled trials in this patient population.

In conclusion, the fight against diabetic HF requires a multidimensional approach, one that integrates systemic metabolic control with targeted molecular interventions. By bridging the gap between mechanistic understanding and clinical application, the next decade holds the potential to transform the management of diabetic HF from symptom control to true disease modification.

## Figures and Tables

**Table 1:** Summary of Randomized Controlled Trials and Real-World Evidence on SGLT2 Inhibitors in Heart Failure.

Name	Method	Result	Conclusion
The DAPA-HF study	N = 4744; Dapagliflozin 10 mg vs. placebo; Follow-up: 18.2 years	Reduced cardiovascular death or HHF by 26% and all-cause mortality by 17% in HFrEF.	Reduces risk of ventricular arrhythmia, cardiac arrest, or sudden death in HFrEF.
The EMPEROR-Reduced study	N = 3730; Empagliflozin 10 mg vs. placebo; Follow-up: 1.5 years	Reduced first cardiovascular death or HF (±CKD) and recurrent HF hospitalizations; slowed eGFR decline.	Empagliflozin use is recommended in HFrEF.
The DELIVER study	N = 6263; Dapagliflozin 10 mg vs. placebo; Follow-up: 2.3 years	Reduced cardiovascular death and HHF by 18%, regardless of LV function.	Reduces risk of primary composite endpoint.
The PRESERVED-HF study	N = 324; Dapagliflozin 10 mg vs. placebo; Follow-up: 12 weeks	Improved symptoms, physical limitations, and 6-min walk test.	Improves symptoms and activity limitations in HFpEF.
The CAMEO-DAPA study	N = 38; NYHA II/III, LVEF > 50%, PCWP; Follow-up: 24 weeks	Resting PCWP ↓ 3.5 mmHg; Exercise PCWP ↓ 6.1 mmHg.	Offers potential benefits for HFpEF.
The EMPEROR-Preserved study	N = 5988; Empagliflozin 10 mg vs. placebo; Follow-up: 26.2 months	Improved cardiovascular death and HHF in HFpEF; unaffected by HR.	Benefit independent of comorbid diabetes.
The EMPA-RESPONSE-AHF study	N = 80; Empagliflozin 10 mg vs. placebo; Follow-up: 60 days	Reduced composite endpoint: worsening HF, HF rehospitalization, death.	Safe and well tolerated in acute decompensated HF.
The EMPA-REG OUTCOME study	N = 7020; Empagliflozin 10 or 25 mg vs. placebo	Reduced 3P-MACE by 14% and CV death by 38%.	Prolonged survival in patients of all ages.
The EMBRACE-HF study	N = 65; Empagliflozin 10 mg vs. placebo; Follow-up: 12 weeks	Reduced mean pulmonary artery diastolic pressure.	Reduced pulmonary artery diastolic pressure.
The EMPULSE study	N = 530; Empagliflozin 10 mg vs. placebo; Follow-up: 90 days	Patients were 36% more likely to have clinical benefit; no heterogeneity.	Clinical benefit within 90 days in acute HF.
The EMPAG-HF study	Empagliflozin 25 mg vs. placebo; Follow-up at discharge & 30 days	No additional renal injury in acute decompensated HF.	Safe and well tolerated.
The VERTIS CV study	N = 8246; Ertugliflozin 5/15 mg vs. placebo; Follow-up: 3.5 years	Non-inferior for 3P-MACE; reduced first and all HF events.	Effect not influenced by baseline HF or LVEF.
The CHIENT-HF study	N = 476; Canagliflozin 100 mg vs. placebo	Improved symptoms regardless of EF or T2DM.	Improves prognosis, symptoms, and quality of life.
CVD-REAL study	Patients from 6 countries; SGLT2i vs. other antidiabetics	Lower HHF and mortality; no heterogeneity between countries.	Associated with reduced HHF and mortality.
Scandinavian registry cohort	Registry data from Denmark, Norway, Sweden; 19% CVD history, 6% HF history	83% dapagliflozin, 16% empagliflozin, 1% canagliflozin; 34% lower HF risk vs. DPP-4i.	Reduced primary HF risk by 34%.
Real-world Taiwan study	N = 12,681 T2DM; dapagliflozin (n = 5812) vs. empagliflozin (n = 6869)	Similar CV event risk, but dapagliflozin reduced HF more than empagliflozin.	Dapagliflozin superior in reducing HF risk.

Abbreviations: HF: heart failure; HHF: hospitalization for heart failure; HfrEF: heart failure with reduced ejection fraction; HfpEF: heart failure with preserved ejection fraction; EF: ejection fraction; LVEF: left ventricular ejection fraction; NYHA: New York Heart Association; PCWP: pulmonary capillary wedge pressure; CKD: chronic kidney disease; T2DM: type 2 diabetes mellitus; 3P-MACE: three-point major adverse cardiovascular events; eGFR: estimated glomerular filtration rate; RCT: randomized controlled trial; NNT: number needed to treat.

**Table 2:** Summary of Antidiabetic Drug Classes and Their Impact on Heart Failure Based on Current Evidence.

Class	Common Medications	Effects in Heart Failure	Clinical Practice Recommendations
SGLT2 inhibitors	Dapagliflozin, Empagliflozin	Beneficial—reduce HHF, cardiovascular death, and CKD progression in HFrEF and HFpEF	Recommended for symptomatic HFrEF and HFpEF patients regardless of DM status; high-quality evidence (Grade A)
GLP-1 RA	Semaglutide	Beneficial—improve symptoms in HFpEF, reduce weight, attenuate remodeling and inflammation	Consider when SGLT2 inhibitors are contraindicated or not tolerated; especially for HFpEF with obesity
DPP-4 inhibitors	Saxagliptin (harmful), Alogliptin (neutral)	Mixed—saxagliptin increases HHF risk; others neutral	Saxagliptin not recommended in T2DM with HF; others with caution
GKA	Dorzagliatin	Potential cardiovascular benefits—improve β-cell function, glycemic control	Promising for CHD and HF, but more RCT data needed before recommendation
Metformin	Metformin	Beneficial—reduces mortality, HF readmissions, improves myocardial metabolism	Recommended for stable T2DM with HF and normal renal function; avoid in acute/decompensated HF
Glycosidase inhibitor	Acarbose	Beneficial—reduces HF risk	Second- or third-line; avoid or discontinue if digoxin is used
Glinides	Repaglinide	Neutral—no increased CV risk	Second- or third-line; monitor closely in HF
Sulfonylureas	Glimepiride	Potentially harmful—increased mortality risk	Not recommended for T2DM with HF
Insulin	Insulin	Potentially harmful—may worsen HF outcomes	Use rapid-/short-acting forms with close monitoring in HF
TZDs	Pioglitazone	Harmful—increase HF and mortality risk	Not recommended for T2DM with HF

Abbreviations: SGLT2, sodium–glucose cotransporter 2; GLP-1 RA, glucagon-like peptide-1 receptor agonist; DPP-4, dipeptidyl peptidase-4; GKA, glucokinase activator; TZD, thiazolidinedione; HFrEF, heart failure with reduced ejection fraction; HFpEF, heart failure with preserved ejection fraction; DM, diabetes mellitus; HF, heart failure; HHF, hospitalization for heart failure; CKD, chronic kidney disease; T2DM, type 2 diabetes mellitus; CHD, coronary heart disease; CV, cardiovascular; RCT, randomized controlled trial.

## Data Availability

No new data were created or analyzed in this study.
